# Criterion Validity of the Yale-Brown Obsessive-Compulsive Scale Second Edition for Diagnosis of Obsessive-Compulsive Disorder in Adults

**DOI:** 10.3389/fpsyt.2018.00431

**Published:** 2018-09-11

**Authors:** Pedro Castro-Rodrigues, Marta Camacho, Sílvia Almeida, Mónica Marinho, Catarina Soares, J. Bernardo Barahona-Corrêa, Albino J. Oliveira-Maia

**Affiliations:** ^1^Champalimaud Clinical Centre, Champalimaud Centre for the Unknown, Lisbon, Portugal; ^2^Champalimaud Research, Champalimaud Centre for the Unknown, Lisbon, Portugal; ^3^NOVA Medical School, Faculdade de Ciências Médicas, Universidade Nova de Lisboa, Lisbon, Portugal; ^4^Centro Hospitalar Psiquiátrico de Lisboa, Lisbon, Portugal; ^5^Department of Psychiatry and Mental Health, Centro Hospitalar de Lisboa Ocidental, Lisbon, Portugal

**Keywords:** Yale-Brown Obsessive-Compulsive Scale—Second Edition (Y-BOCS-II), obsessive-compulsive disorder, psychometric properties, criterion validity, Portuguese language

## Abstract

**Background:** While the Yale-Brown Obsessive-Compulsive Scale Second Edition (Y-BOCS-II) is the gold-standard for measurement of obsessive-compulsive (OC) symptom severity, its factor structure is still a matter of debate and, most importantly, criterion validity for diagnosis of OC disorder (OCD) has not been tested. This study aimed to clarify factor structure and criterion validity of the Y-BOCS-II.

**Methods:** We first validated and quantified the psychometric properties of a culturally adapted Portuguese translation of the Y-BOCS-II (PY-BOCS-II). The PY-BOCS-II and other psychometric instruments, including the OCD subscale of the Structured Clinical Interview for the DSM-IV, used to define OCD diagnosis, were administered to 187 participants (52 patients with OCD, 18 with other mood and anxiety disorders and 117 healthy subjects). In a subsample of 20 OCD patients and the 18 patients with other diagnoses, PY-BOCS-II was applied by clinicians blinded to diagnosis.

**Results:** PY-BOCS-II had excellent internal consistency (Cronbach's α = 0.96) and very good test-retest reliability (Pearson's *r* = 0.94). Exploratory factor analysis revealed a two-factor structure with loadings consistent with the Obsessions and Compulsions subscales, and there was good to acceptable convergent and divergent validity. Importantly, the area under the curve (AUC) of the receiver operating characteristic (ROC) curve suggested elevated accuracy in discriminating between patients with OCD and control subjects (AUC = 0.96; 95% confidence interval [CI]: 0.92–0.99), that was retained in comparisons with age, gender and education matched controls (AUC = 0.95; 95% CI: 0.91–0.99), as well as with patients with other mood and anxiety disorders (AUC = 0.93; 95% CI: 0.84–1). Additionally, a cut-off score of 13 had optimal discriminatory ability for the diagnosis of OCD, with sensitivity ranging between 85 and 90%, and specificity between 94 and 97%, respectively when all samples or only the clinical samples were considered.

**Conclusion:** The PY-BOCS-II has excellent psychometric properties to assess the severity of obsessive-compulsive symptoms, reflecting obsessive, and compulsive dimensions, compatible with currently defined subscales. Furthermore, we found that a cut-off of 13 for the Y-BOCS-II total score has good to excellent sensitivity and specificity for the diagnosis of OCD.

## Introduction

Obsessive-compulsive disorder (OCD) is a chronic and incapacitating neuropsychiatric condition, with a lifetime prevalence of 2.3% in the United States and an estimated prevalence of 5.3% in Portugal (1–3). It is characterized by the presence of obsessions (recurrent and persistent thoughts, experienced as intrusive or inappropriate and causing marked anxiety or distress) and/or compulsions (repetitive behaviors or mental acts that the person feels driven to perform, typically to reduce the anxiety caused by the obsessions) (4–7). Accurate assessment of OCD is critical due to its under-diagnosis, difficulty in establishing accurate diagnosis and need for careful and specific treatment planning and evaluation (8).

The Yale-Brown Obsessive-Compulsive Scale (Y-BOCS) is a clinician-administered instrument, developed in 1989 to assess the presence and severity of obsessive-compulsive symptoms (9, 10). It is divided into a symptom checklist and a severity scale. The symptom checklist comprises 54 dichotomous items assessing current or prior presence of specific obsessions and compulsions. The severity scale consists of 10 items that quantify the impact of obsessions and compulsions identified using the symptom checklist. These 10 items are 5-point Likert-type scales characterizing the time spent on compulsions (item 1), interference from obsessions (item 2), distress associated with obsessions (item 3), resistance to obsessions (item 4), subject's control over obsessions (item 5) and equivalent items for compulsions (items 6–10). The Y-BOCS has shown good psychometric properties and sensitivity to the therapeutic effects of medication and psychotherapy (9–14). However, several problems have been identified for this scale, including a poor conceptual fit of the “resistance to obsessions” item, possibly contributing toward inconsistent factor structure, with some studies finding a two-factor (obsessions and compulsions) and others a three-factor structure (obsessions, compulsions, and resistance to obsessions), as well as low sensitivity to change in severe cases and poor divergent validity relative to depressive symptoms (15–20).

To address some of these problems, a revised version, the Y-BOCS-II, was published in 2000 (20), with several differences relative to the original scale. Specifically, the obsessions and compulsions checklists are not formally subdivided into different symptom groups, some items in the symptom checklist were reworded and expanded, and a new checklist for avoidance was created. Additionally, in the severity scale, the item assessing “resistance against obsessions” was replaced by an item of “obsessions-free interval,” the scoring for each item was revised from 0–4 to 0–5, and the order of assessment of items was changed. Furthermore, avoidance was considered in the definition of severity, namely for the items of interference from obsessions and interference from compulsions. Finally, the definitions of obsessions and compulsions were rephrased and several ancillary items removed from the text. Y-BOCS-II has excellent psychometric properties, with strong internal consistency, high test-retest and interrater reliabilities, and strong correlations with other clinician-rated measures of obsessive-compulsive symptom severity, namely the National Institute of Mental Health Global Obsessive Compulsive Scale (NIMH-GOCS), and only moderate correlations with measures of worry and depressive symptoms (20). The authors of the original scale also conducted an exploratory factor analysis, the results of which were consistent with the obsession and compulsion severity subscales (20). Thus, the Y-BOCS scales are typically considered the gold-standard instrument in assessing severity of obsessive-compulsive symptoms (8, 21), with the Y-BOCS-II translated and validated for other languages in addition to English (22, 23).

Further exploration of the psychometric properties of the Y-BOCS-II is pertinent for several reasons. In fact, criterion validity of this scale has not been tested, namely through comparisons between OCD patients and control samples, such as healthy subjects or, most importantly, other patients with similar disorders. Such comparisons would be important to define a cut-off value, allowing clinicians to establish that obsessive or compulsive symptoms may reflect an OCD diagnosis, rather than symptoms of a mood or anxiety disorder (e.g., rumination in depressive disorders and fear or worries in anxiety disorders) (24). Furthermore, the underlying factor structure of the Y-BOCS-II is still a matter of debate (21), with the original American and the Thai versions showing a two-factor structure, as described above, while the Italian version has a different factor structure, with distinct dimensions (20, 22, 23). Finally, the temporal stability of the Y-BOCS-II, while clinically relevant to understand temporal stability for longer periods (20, 22, 25), has only been tested in short intervals, no longer than 2 weeks.

Here, we explored the psychometric properties of a culturally adapted Portuguese translation of the Y-BOCS-II (PY-BOCS-II), including internal consistency, factor structure, test-retest reliability, convergent validity, and divergent validity. Importantly, we focused on the scale's criterion validity, through comparisons of total scores between patients with OCD and control subjects, including both healthy volunteers and patients with other mood and anxiety disorders, as defined by a gold-standard instrument for diagnosis of OCD.

## Materials and methods

### Participants

Eligibility was assessed in 223 participants, recruited either at the Champalimaud Clinical Centre or Centro Hospitalar Psiquiátrico de Lisboa. Patients with a clinical diagnosis of OCD (*n* = 60) were referred to the study by attending psychiatrists and psychologists, while control patients with other psychiatric diagnoses (*n* = 35) were selected randomly from the institutional databases at each institution. A convenience sample of 128 healthy community dwelling subjects was also recruited at each of the two institutions. Exclusion criteria for all samples were: acute medical illness; active neurological disease or clinically significant focal structural lesion of the central nervous system; acute episode of neuropsychiatric disease requiring hospitalization; history or clinical evidence of chronic psychosis, dementia, developmental disorders with low intelligence quotient or any other form of cognitive impairment; current substance or alcohol abuse or dependence; and illiteracy or otherwise not understanding the study's instructions. For all participants except those in the OCD sample, current diagnosis of OCD, as assessed by structured diagnostic interviews (OCD subscale of the Structured Clinical Interview for the DSM-IV and MINI Neuropsychiatry Interview), was also an exclusion criterion. For the healthy volunteers sample, current or past history of any psychiatric disorder, as assessed by the MINI Neuropsychiatry Interview, was an additional exclusion criterion. Among the 223 participants that were assessed, 52 OCD patients, 18 patients with non-OCD mood or anxiety disorders and 117 healthy participants were eligible for the study.

### Measures

#### Y-BOCS-II

The Y-BOCS-II consists of two main components: a 67-item Symptom Checklist and a 10-item Severity Scale (20). In the Symptom Checklist, 29 items assess the presence of specific obsessions, another 29 items assess the presence of specific compulsions, and the remaining 9 items assess the presence of avoidance. Each item is dichotomously rated for current (i.e., within the past month) and past presence. In the Severity Scale, items assess, for the previous week, time spent with either obsessions or compulsions (items 1 and 6, respectively), obsession-free interval (item 2), resistance to compulsions (item 7), degree of control over either obsessions or compulsions (items 3 and 8, respectively), distress associated either with obsessions or with the impossibility of performing compulsions (items 4 and 9, respectively), and interference from either obsessions or compulsions (items 5 and 10, respectively). Items 5 and 10 also assess severity of avoidance related with obsessions or compulsions, respectively. Each of the 10 items is rated in a 6-point scale (0–5) and 2 subscales are typically considered: an Obsessions subscale (items 1–5) and a Compulsions subscale (items 6–10). A more detailed description of the scale is given in the Introduction.

The Y-BOCS-II was not previously validated for use in adult populations speaking European Portuguese. To guarantee that linguistic and semantic equivalence of the Y-BOCS-II was preserved for use in such populations, we used a 3-step translation/back-translation method to obtain a Portuguese Y-BOCS-II (PY-BOCS-II). For the first step, multiple independent translations from US English into European Portuguese, performed separately by four bilingual experts in Psychology or Psychiatry of Portuguese dominant language, were obtained, and then joined into a single consensus translation by the 4 translators. In the second step, back-translation of the consensus Portuguese translation into English was performed by two bilingual translators, of English dominant language, that were not involved in the original translation. This was followed by comparison of the back-translated versions by the original translation team, for creation of a consensus back-translation. In the last step, the consensus back-translation was compared with the original version by the initial translation team, and also sent for review and comments by the original authors of the Y-BOCS-II. This allowed for adjustments of the consensus Portuguese translation, to obtain a refined consensus Portuguese translation of the Y-BOCS-II. This version was then discussed among a panel of Portuguese-speaking experts in the fields of Psychiatry or Psychology, including but not limited to the original translation team, for assessment of face validity and proposal of additional adjustments for cultural adaptation. Finally, the scale was applied to a group of 10 patients suffering from OCD, followed by interviews for qualitative assessment of duration, cognitive effort, and adequate comprehension of items. Considering the input from these patients, the translation was further adapted, and the final version of the PY-BOCS was defined.

#### Structured clinical interview for the DSM-IV, OCD subscale (SCID-OCD)

The OCD Subscale of the SCID-IV is a semi-structured interview that allows for the diagnosis of current OCD according to DSM-IV criteria (26). It has been validated for Brazilian Portuguese by Del-Ben et al. (27) and we adapted this version for European Portuguese. The SCID-OCD was used to discriminate between participants with and without OCD, for the purpose of criterion validity assessment.

#### Mini neuropsychiatric interview

The MINI is a brief structured clinical interview divided into 15 modules (28). It allows for detection of major depressive disorder (MDD), dysthymia, suicide risk, manic and hypomanic episodes, panic disorder, agoraphobia, social phobia, generalized anxiety disorder (GAD), OCD, post-traumatic stress-disorder, alcohol abuse or dependence, substance abuse or dependence, psychotic disorders, anorexia nervosa and bulimia nervosa, based on the rapid screening of DSM-IV diagnostic criteria. The interview has been translated to European Portuguese by Guterres, Levy and Amorim (29). We used this version of the MINI to assess comorbidity and identify exclusion criteria.

#### Beck depression inventory II (BDI-II)

The BDI-II is a 21-item self-report screening instrument that assesses the presence of depressive symptoms in the previous 15 days (30). Responses are scored from 0 (“absent”) to 3 (“severe”). It was validated to the Portuguese adult population by Campos and Gonçalves (31). We used the BDI-II results to assess divergent validity with the PY-BOCS-II.

#### State-trait anxiety inventory (STAI)

The STAI is a widely-used 40 item self-report screening instrument that assesses the presence of anxiety symptoms (32). It is composed of two subscales: the STAI-state and the STAI-trait. Trait anxiety corresponds to feelings of tension, apprehension and increased autonomic activity and is a relatively stable personality trait (32, 33). People with high trait anxiety have a tendency to perceive more situations as dangerous or threatening than people who have lower trait anxiety scores. State anxiety, on the other hand, fluctuates over time according to the presence of stressors. Individuals with high trait anxiety scores also tend to have higher state anxiety scores (32, 33). The scale was validated for use in Portuguese-speaking adults by Santos and Silva (34).

#### Coimbra obsessive inventory (COI—inventário obsessivo de coimbra)

The COI is a self-report scale, developed for the Portuguese population, that assesses obsessive and compulsive symptoms through 12 dimensions, namely doubt and indecision, intrusive thoughts and covert rituals, magical thinking, slowness and repetition, need for control, need for order and symmetry, collection and hoarding, religious obsessions and compulsions, somatic obsessions, and obsessive and aggressive impulses (35). It is subdivided in “frequency” and “emotional distress” subscales. The COI score was used to assess convergent validity for the PY-BOCS-II.

### Procedures

Study procedures and protocol were reviewed and approved by the Ethics Committees of the Champalimaud Centre for the Unknown and of Centro Hospitalar Psiquiátrico de Lisboa. All subjects gave written informed consent in accordance with the Declaration of Helsinki. In the non-blinded sample, after participants had responded to a global clinical questionnaire, instruments were applied in the following order: MINI, SCID-IV, PY-BOCS-II, BDI-II, STAI, COI. In the blinded sample, in a first session participants responded to the clinical questionnaire and the following instruments were applied, in the same order: MINI, SCID-IV, BDI-II, STAI, COI. In a second session, conducted by another researcher who did not have access to the first set of results, PY-BOCS-II was applied. Temporal stability was tested in a subsample of 27 OCD patients and 72 healthy participants by applying PY-BOCS-II a second time, 4 weeks after initial testing.

### Data analysis

Descriptive statistics were calculated for sociodemographic and psychometric data, including means and standard deviations, minimum and maximum absolute values and percentage. We used independent samples *t*-tests to compare means between groups, except for gender (in which chi-squared was used), with two-tailed significance values and the alpha-level was set to 0.05. We assessed several psychometric properties of the PY-BOCS-II. To estimate reliability, we analyzed internal consistency using Cronbach's α and temporal stability using Pearson's correlation coefficient. To assess dimensionality, exploratory factor analysis with principal axis factoring and oblique rotation was performed in the Severity Scale. Factor analysis of the Symptom Checklist was not performed due to insufficient sample size of the OCD sample (a sample size of 5–10 participants per item is generally recommended—for 67 items a much larger sample size would be needed) (36). To assess construct validity, we used Pearson's correlation coefficient of PY-BOCS-II scores with COI scores for convergent validity, and with BDI-II scores and STAI scores for divergent validity. Finally, criterion validity was analyzed by studying the relationship between PY-BOCS-II scores and SCID-OCD classification using receiving operating characteristic (ROC) curves. Such curves are obtained by plotting the true positive rate (i.e., sensitivity) in function of the false positive rate (1-specificity), with each point in the curve representing a sensitivity/specificity pair corresponding to each possible decision threshold. Here, the area under the curve (AUC) of the ROC curve reflects the probability that a randomly chosen individual with OCD had a higher PY-BOCS-II score than a randomly chosen individual without OCD diagnosis (with diagnosis defined by the SCID-OCD) The decision threshold, or cut-off value, for OCD diagnosis was then chosen according to the ROC curve, as the total score that maximized sensitivity and specificity over all possible values.

## Results

### Descriptive statistics

Sociodemographic data and mean scores of all psychometric instruments are presented in Table 1. While the non-OCD sample was slightly younger than the OCD sample, there were no significant differences in gender or education. A more detailed subgroup analysis revealed a more complex pattern of differences between subgroups (see Table S1). In the OCD sample, the most common comorbid diagnoses were MDD (38%), GAD (17%), prior MDD (15%), panic disorder (15%), and social phobia (12%). For the mood and anxiety disorders sample, a full description of diagnoses is listed in Table S2 and included MDD, dysthymia, Bipolar Disorder (BD), GAD, and panic disorder. Descriptive statistics of individual PY-BOCS-II Severity Scale items in the OCD sample are presented in Table 2. The PY-BOCS-II total score had a weak positive correlation with age (*r* = 0.28) when considering all participants, but in OCD patients this correlation was non-significant. Also, across all participants, there were no statistically significant differences between genders in any of the psychometric measures (*t* < 1.23; *p* > 0.21), and the correlations with education were either non-significant (for the PY-BOCS-II total score) or weak (*r* < 0.3 for all other psychometric measures).

**Table 1:** Sociodemographic and psychometric data from each sample.

	**OCD Sample (*****n*** = **52)**		**Non-OCD Sample (*****n*** = **135)**		
	**Range**	**Mean (SD)**	**Range**	**Mean (SD)**	***p*-value**
Gender (% male)	42.3%		30%		0.1
Age (years)	19–62	40.0 (10.0)	20–64	32.9 (9.5)	<0.001
Education (years)	7–23	14.7 (3.4)	4–23	15.4 (3.3)	0.2
Y-BOCS-II total score	0–45	22.7 (10.4)	0–25	1.8 (3.9)	<0.001
BDI-II total score	1–45	22.2 (13.6)	0–42	6.2 (9.1)	<0.001
STAI-state score	22–75	47.9 (14.9)	20–75	33.8 (10.8)	<0.001
STAI-trait score	26–77	56.9 (14.4)	20–74	32.8 (10.7)	<0.001
COI total score	18–332	137.9 (82.7)	0–290	31.9 (40.0)	<0.001

*For all variables, mean and standard deviation are shown, except for gender (presented as percentage of males). Differences were tested using chi-square for gender and independent samples t-test for the other variables (p-values displayed). OCD, Obsessive-compulsive disorder; Y-BOCS-II, Yale-Brown Obsessive-Compulsive Scale-II; BDI-II, Beck Depression Inventory-II; STAI, State-Trait Anxiety Inventory; COI, Coimbra Obsessive Inventory*.

**Table 2:** Individual Y-BOCS-II item summaries for the OCD sample.

**Items**	**Statistics**			**Percentage of endorsement**							**Reliability**	
	**Mean (SD)**	**Sk**	**Ku**	**0**	**1**	**2**	**3**	**4**	**5**	**Total**	**Item-total corr**.	**α if deleted**
1	2.0 (1.3)	0.7	0.2	9.6	30.8	30.8	17.3	3.8	7.7	100	0.65	0.87
2	2.4 (1.5)	0.0	−1.0	11.5	21.2	21.2	19.2	23.1	5.8	100	0.58	0.87
3	2.6 (1.6)	−0.3	−1.0	15.4	11.5	15.4	23.1	23.1	11.5	100	0.63	0.87
4	2.2 (1.3)	0.2	−0.3	9.6	21.2	26.9	28.8	7.7	5.8	100	0.50	0.88
5	1.8 (1.4)	0.5	−0.5	19.2	26.9	26.9	11.5	11.5	3.8	100	0.64	0.87
6	1.9 (1.3)	0.5	−0.1	13.5	30.8	25.0	23.1	3.8	3.8	100	0.64	0.87
7	2.5 (1.7)	−0.2	−1.3	21.2	11.5	11.5	19.2	25.0	11.5	100	0.53	0.88
8	2.8 (1.6)	−0.5	−0.7	13.5	5.8	19.2	19.2	30.8	11.5	100	0.68	0.87
9	2.7 (1.4)	−0.1	−0.7	7.7	13.5	25.0	25.0	17.3	11.5	100	0.69	0.87
10	1.8 (1.5)	0.2	−1.1	26.9	19.2	17.3	23.1	11.5	1.9	100	0.65	0.87

*For each item of the Y-BOCS-II, the mean, standard deviation and the percentage of endorsement for each possible item score (range 0-5) is displayed. OCD, Obsessive-compulsive disorder; Y-BOCS-II, Yale-Brown Obsessive-Compulsive Scale-II; SD, Standard deviation; comp, compulsions; Sk, Skewness; Ku, Kurtosis; Item-total corr, Item-total correlation; α if deleted, Cronbach's α if item is deleted*.

### Reliability

A Cronbach's alpha of 0.96 was obtained for the PY-BOCS-II severity scale when data from all participants were considered, demonstrating robust internal consistency. A slightly lower value (0.94) was found for both the Obsessive and Compulsive subscales, when tested separately. Furthermore, Cronbach's alpha remained stable with removal of any item from the scale (0.96 for all items), and corrected item-total correlations ranged between 0.8 and 0.87. Reliability measures when considering only data from the OCD sample are presented in Table 2.

Regarding temporal stability, assessed in 99 participants in the global sample, a Pearson's r of 0.94 (*p* < 0.001) was obtained for the correlation of PY-BOCS-II total score at the first application and 30 days later. When considering only the OCD sample (*n* = 27), test-retest reliability was slightly higher (*r* = 0.95, *p* < 0.001). Finally, the temporal stability of the Obsessions subscale was higher than the temporal stability of the Compulsions subscale, both when considering all participants (*r* = 0.94 vs. *r* = 0.89, respectively) and OCD patients only (*r* = 0.92 vs. *r* = 0.84, respectively).

### Dimensionality

**Figure 1:**
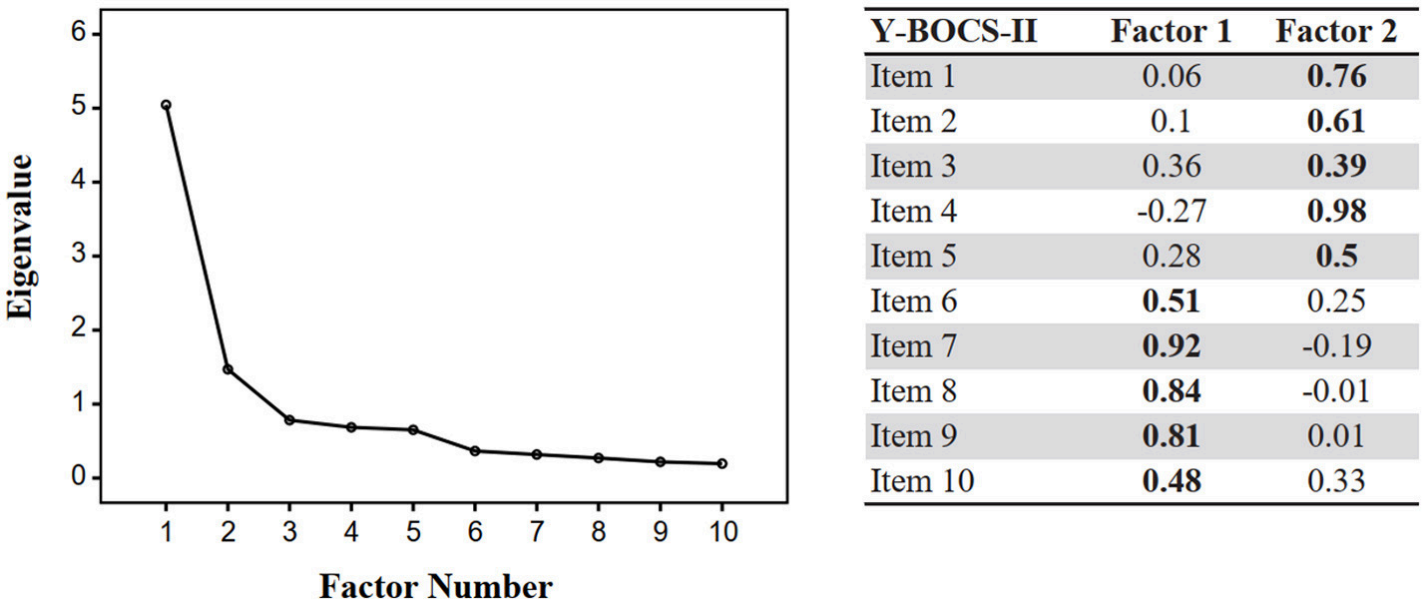
Scree plot (exploratory factor analysis) and pattern matrix for Y-BOCS-II factors in the OCD sample. In the pattern matrix, standardized weights of a regression analysis in which item responses are predicted from their levels of the underlying factors are represented. Factor loadings above 0.4 or highest factor loading shown in bold.

We conducted exploratory factor analysis using principal axis factoring with promax rotation in the OCD sample. The Kaiser-Meier-Olkin measure of sample adequacy was 0.836, above the recommended value of 0.6, and the Bartlett's test of sphericity was significant [*X*${}_{\left(45\right)}^{2}$ = 265.75, *p* < 0.001]. Two factors with eigenvalues >1 were obtained (eigenvalues of 5.05 for the first factor and 1.47 for the second factor) and this two-factor solution was consistent with the deflection of the scree plot (Figure 1). The pattern matrix revealed that items 6–10 had higher loadings on factor 1 (all >0.4) and items 1–5 on factor 2. Item 3 had relatively small loadings on both factors, although with slightly higher loading on factor 2. The correlation between factor 1 and factor 2 was 0.55.

### Construct validity

Measures of construct validity, using correlations between the PY-BOCS-II and several self-report psychometric measures, are shown in Table 3. For convergent validity, we found a significant and strong correlation between the PY-BOCS-II total score and the score for a self-report obsessive-compulsive inventory (COI) (*r* = 0.67, *p* < 0.001), with similar correlations with each of the COI subscales (*r* = 0.67 for the frequency subscale and *r* = 0.66 for the emotional distress subscale, both with *p* < 0.001). For divergent validity, the correlations between the Y-BOCS-II total score and the STAI-state scores was only moderate (*r* = 0.43, *p* < 0.001), higher, but still moderate, for the BDI-II score (*r* = 0.57, *p* < 0.001), and strong for the STAI-trait (*r* = 0.68, *p* < 0.001). Furthermore, the PY-BOCS-II Compulsions subscale had lower correlation with BDI-II and both STAI scores than the Obsessions subscale (Table 3) suggesting a better divergent validity for the Compulsions subscale. Table S3 shows the correlation matrix for all psychometric measures in all participants.

**Table 3:** Correlations between psychometric measures and Y-BOCS-II partial and total score in all participants.

**All participants**	**Y-BOCS-II obsessions**	**Y-BOCS-II compulsions**	**Y-BOCS-II total**
COI total	0.67	0.64	0.67
BDI-II total	0.61	0.48	0.57
STAI-state	0.46	0.37	0.43
STAI-trait	0.73	0.58	0.68

*Pearson's product moment correlation coefficient used as correlation measure. All correlations are highly significant (p's < 0.001). Y-BOCS-II, Yale-Brown Obsessive-Compulsive Scale-II; COI, Coimbra Obsessive Inventory; BDI-II, Beck Depression Inventory-II; STAI, State-Trait Anxiety Inventory*.

### Criterion validity

**Figure 2:**
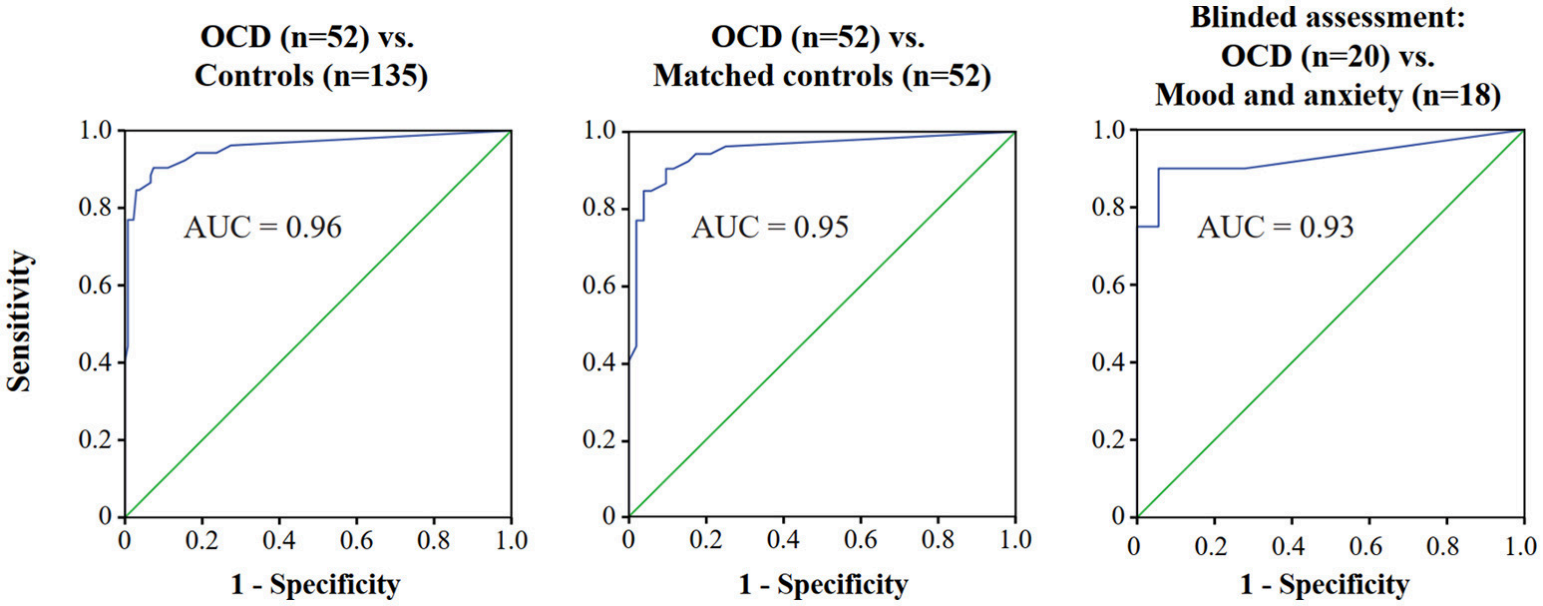
ROC curves for use of the Y-BOCS-II to identify OCD. Plot of the true positive rate (1—specificity) against the false positive rate (sensitivity) for the different possible cut-offs of the Y-BOCS-II using the SCID-OCD as the diagnostic instrument. In the left panel, all participants were considered. In the middle panel, OCD and age-, gender-, and education-matched controls (balanced mixture of healthy subjects and patients with mood and anxiety disorders) are considered. In the right panel, patients who completed a blinded assessment are considered. ROC, Receiver operating characteristic; Y-BOCS-II, Yale-Brown Obsessive-Compulsive Scale-II; OCD, Obsessive-compulsive disorder; AUC, Area under the curve.

To assess criterion validity, we created receiver operating characteristic (ROC) curves (Figure 2), using the SCID-OCD as the discriminator between participants with OCD (*n* = 35) and controls (*n* = 135; Figure 2 left panel). An area under the curve (AUC) of 0.96 (95% Confidence interval [95% CI]: 0.92, 0.99) was obtained, and further analyses of the ROC curve values showed that a PY-BOCS-II total score of 13 points, when used as a cut-off for diagnosis, correctly identifies OCD with a sensitivity of 85% and a specificity of 97% (Table 4). To further explore the discriminatory capacity of the Y-BOCS-II, a similar analysis was performed comparing the OCD sample with a group of age-, gender- ,and education-matched controls (frequency-matched balanced mixture of healthy subjects and patients with mood and anxiety disorders; Figure 2 middle panel). The AUC was similar (AUC = 0.95; 95% CI: 0.91, 0.99) and a total score cut-off of 13 points remained optimal, with sensitivity of 85% and specificity of 96%. Importantly, the same analyses were repeated in data from a subgroup of patients with either OCD (*n* = 20) or other mood and anxiety disorders (*n* = 18), for whom PY-BOCS-II was applied by a researcher blinded to diagnosis and to the results of other psychometric tests. In this group (Figure 2 right panel), AUC was only slightly lower (AUC = 0.93; 95% CI: 0.84, 1) and the 13-point cut-off resulted in sensitivity of 90% and specificity of 94% for diagnosis of OCD.

**Table 4:** Coordinates for the ROC curve of the Y-BOCS-II using all participants.

**Y-BOCS-II cut-off score**	**Sensitivity (%)**	**Specificity (%)**
0.50	96.2	72.6
1.50	94.2	76.3
2.50	94.2	78.5
3.50	94.2	81.5
4.50	92.3	84.4
5.50	90.4	88.9
6.50	90.4	91.1
7.50	90.4	91.9
8.50	90.4	92.6
9.50	88.5	93.3
10.50	86.5	93.3
11.50	84.6	96.3
**13.00**	**84.6**	**97.0**
14.50	76.9	97.8
15.50	76.9	98.5
16.50	76.9	99.3
17.50	75.0	99.3
18.50	73.1	99.3
19.50	67.3	99.3
20.50	63.5	99.3
21.50	59.6	99.3
22.50	55.8	99.3
23.50	50.0	99.3
24.50	44.2	99.3
25.50	40.4	100.0
		

*For each coordinate (Y-BOCS-II total score), its sensitivity and specificity in identifying OCD are shown. The total score with the best sensitivity and specificity for diagnosis of OCD is shown in bold. ROC, Receiver operating characteristic; Y-BOCS-II, Yale-Brown Obsessive-Compulsive Scale-II; OCD, Obsessive-compulsive disorder*.

## Discussion

Here, we have translated and successfully validated the Y-BOCS-II for the Portuguese adult population. A translated and culturally adapted version of the scale had excellent reliability and was valid for assessment of the severity of obsessive-compulsive symptoms. Our results further supported a two-factor structure for the scale, consistent with the Obsessions and Compulsions subscales proposed by the original authors. Importantly, and addressing the main objective of this study, we have demonstrated, to the best of our knowledge for the first time, that the Y-BOCS-II adequately discriminates patients with OCD, and that a cut-off of 13 points for the Y-BOCS-II total score has excellent sensitivity and specificity for that diagnosis.

Our results on reliability of the PY-BOCS-II are in line with the studies that have previously assessed the psychometric properties of this scale. Storch and colleagues found strong internal consistency (Cronbach's alpha = 0.89), similar to what was described later for the Thai (0.94) and Italian (0.83) versions of the scale (20, 22, 23). Regarding test-retest reliability, high values were reported in the original description of the psychometric properties of the scale (Intraclass correlation [ICC]>0.85), as well as for the Italian version (ICC = 0.74), while the Thai version did not assess this psychometric dimension (20, 22, 23). Recently, psychometric properties of the original American version of the Y-BOCS-II were retested, with findings of good internal consistency (Cronbach's alpha = 0.86), acceptable test-retest reliability (*r* = 0.64–0.81) and excellent inter-rater reliability (ICC = 0.97–0.99) (25). Our findings for internal consistency (Cronbach's alpha = 0.96) and test-retest reliability (*r* = 0.94–0.95) are in the upper range of prior studies, suggesting that the process of translation and cultural adaptation was successful. Furthermore, other authors have suggested that temporal stability be tested with longer test-retest intervals than 2 weeks (20, 22, 25). Ours is, to our knowledge, the first study to demonstrate stability of test scores after 4 weeks.

Regarding dimensionality, and due to lack of consensus regarding the factor structure of the Y-BOCS-II, we decided to perform an exploratory factor analysis rather than a confirmatory factory analysis, as was common practice in previous studies. While, in general terms, our results replicate previous findings of a two-factor solution corresponding to obsessions and compulsions, there a few subtle but noteworthy differences (20, 23). Specifically, for the original and Thai versions of the task, interference from obsessions (item 5) had high loadings (>0.4) on both factors, with the authors of the Thai version also reporting higher loadings of distress associated with obsessions (item 4) on the compulsions factor than the obsessions factor (23). Loadings in our data were more clearly distributed between the two factors, with the first five items mainly loading on a factor that is consistent with an Obsessive dimension, and the last five items loading mainly on the second factor, consistent with a Compulsive dimension. Unexpectedly, item 3 (“control over obsessions”) loaded similarly on both factors, possibly because a subset of patients may feel that their level of control over obsessions is dependent on the frequency and severity of compulsions. Importantly, our results are in marked contrast with those for the Italian version of the scale, which revealed a “symptom severity” factor (items 1–4 and 6–9) and “interference from symptoms in daily life” factor (items 5 and 10) (22). It is unclear whether these differences in factor structure reflect true cultural differences across different countries with respect to the presentation of OCD, or are merely due to methodological differences, namely regarding sample size.

With regards to convergent validity, the PY-BOCS-II showed a correlation of 0.67 with self-reported obsessive-compulsive symptom scores in the COI. This correlation was observed even though a high score in the COI reflects a high number of different symptoms causing distress, but not necessarily the severity of individual symptoms (9, 37), while the Y-BOCS-II measures severity of OCD symptoms regardless of the number of different symptoms. Other authors have found low to moderate correlations between Y-BOCS-II scores and scores on self-reported OCD symptom assessment tools such as the Obsessive-Compulsive Inventory-Revised (OCI-R), while correlations with clinician-rated obsessive-compulsive symptom scales such as the National Institute of Mental Health Global Obsessive Compulsive Scale are stronger (e.g., *r* = 0.85) (20). Assessing convergent validity against a clinician-rated scale would thus, in all likelihood, have yielded a more robust correlation for the PY-BOCS-II. For divergent validity, the PY-BOCS-II total score showed a moderate correlation with both depression and state-anxiety scores, and a strong correlation with trait-anxiety scores. This observation replicates the findings of previous studies on the psychometric properties of the Y-BOCS, that also found weak correlations with self-reported measures of anxiety and moderate to strong correlations with self-reported measures of depression such as the Inventory of Depressive Symptomatology—Self-Report (*r* = 0.35) (20), the Patient Healthy Questionnaire (*r* = 0.45) (23), the BDI (*r* = 0.40) (22), or the Depression Anxiety Stress Scale—Depression subscale (*r* = 0.41) (25). Together, the currently available data suggests that divergent validity regarding depression symptoms is, at best, only moderate. This was also a problem with the first version of the Y-BOCS, and may be related to the high co-morbidity between OCD and major depressive disorder (MDD), which may be as high as 50% (38–40). As to the robust correlation between the PY-BOCS-II and STAI-trait anxiety, it may simply reflect the fact that patients with more severe OCD tend to have higher levels of longstanding comorbid anxiety, rather than a true limitation in the scale's ability to discriminate between these two dimensions.

Our findings of higher correlations with self-reported depression and anxiety symptoms in the Obsessions subscale than in the Compulsions subscale suggest that the latter may have better divergent validity. This finding is in line with the results from Storch and colleagues. In their study, the Y-BOCS-II Compulsion subscale had higher correlations with the NIMH-GOCS and with the OCI-R and lower correlations with the PSWQ and with the IDS-SR when compared with the Obsessions subscale (20). For the Thai version of the Y-BOCS-II, the correlations of subscales with depressive symptoms were non-significant and in the Italian version they were not presented (22, 23). This finding is particularly interesting because it could suggest higher tendency for obsessions than for compulsions in patients with comorbid OCD and MDD and higher tendency for compulsions in patients with OCD only.

The main objective of this project, however, was to clarify criterion validity for this scale. AUC of the ROC curves demonstrated that the Y-BOCS is accurate in discriminating between patients with OCD and others without the disorder. To our knowledge, this is the first study exploring criterion-related validity of the Y-BOCS-II in OCD patients, healthy controls and patients with other psychiatric disorders. The cut-off value that we propose (Y-BOCS total score = 13) is in line with previous findings using the first edition of the Y-BOCS (14). Using that version, Farris and colleagues have shown that a posttreatment YBOCS score of 14 or lower was the best predictor of symptom remission and that a posttreatment YBOCS score of 12 or lower was the best predictor of wellness (defined as symptom remission, good quality of life and high level of adaptive functioning) (14). However, it is important to note that this study focused on treatment response and that while the first edition of the YBOCS has an upper limit of 40 points, the upper limit of Y-BOCS-II is 50 points. In any case, the cut-off we propose here can be useful from a diagnostic perspective, because clinicians often assess patients with obsession-like ideas or compulsive-like behaviors, who may or may not suffer from OCD.

Nevertheless, our study is not free of limitations. Regarding validation of the PY-BOCS-II, information about inter-rater reliability would be reassuring. However, all previous studies of psychometric measures of the Y-BOCS-II which have performed this analysis have found excellent inter-rater reliability (20, 22, 25). Furthermore, it would have been desirable to have larger sample sizes, namely in the non-OCD clinical control group, as well as to have a control group without significant differences in demographic characteristics, especially considering the weak positive correlations with age across all psychometric instruments used. However, the Y-BOCS-II had the weakest correlations with age and, in the OCD group, the correlation between Y-BOCS-II and age was non-significant. Nevertheless, to eliminate any potential effects of such differences in the ROC curves, we selected a sample of age-, gender- and education-matched controls and repeated our main analysis only with this group, obtaining confirmation of our previous results. Also, in a subsample of individuals (32 OCD participants), raters were not blind to diagnosis, which could lead to criterion contamination. To account for this potential limitation, we also created ROC curves using only the subset of OCD and non-OCD patients that were assessed in a blinded fashion. While the number of participants included in this analysis was lower, the results obtained were very similar to the remaining ROC curves, thus validating our findings. In the future it could be important to repeat this specific analysis using larger OCD and non-OCD clinical samples. The use of the SCID-OCD as a diagnostic instrument can also be considered a limitation because it has never been validated for the Portuguese population. However, it has been validated for Brazilian Portuguese and the adaptation to European Portuguese was very straightforward. Furthermore, it must be noted that the version of SCID used here was according to DSM-IV, and thus includes hoarding symptoms, which are considered a separate disorder in DSM-5 (Hoarding Disorder). However, we do not believe that this had a significant impact in our results, since none of the participants included in the study presented exclusively hoarding symptoms, as assessed by the Y-BOCS-II Symptom Checklist (items 26 and 46). Finally, future studies could address the properties of the Y-BOCS-II regarding classification of treatment sensitivity, as has been done for the first version of the scale.

In conclusion, we have successfully translated and validated the Y-BOCS-II for use in the Portuguese adult population, showing that the Portuguese version of the Y-BOCS-II maintains the psychometric properties of the original version in evaluating the severity of obsessive-compulsive symptoms. Using this version of the task we have also, for the first time, assessed criterion validity of the Y-BOCS-II, by exploring its capacity to distinguish between patients with OCD and subjects in several clinical and non-clinical groups, using both a blinded and a non-blinded design. Our results suggest that a Y-BOCS-II total score cut-off of 13 has good sensitivity and excellent specificity in identifying OCD. Although a replication in a larger sample, with a blinded study design, would be important to confirm our findings, these results are useful given the importance of correctly assessing obsessive-compulsive symptoms to establish an adequate diagnosis and a thorough treatment plan.

## Author contributions

PC-R, MC, JB-C, and AO-M conceived and designed the study. PC-R, MC, JB-C, and AO-M participated in the translation procedures. PC-R, MC, SA, MM, and CS collected data. PC-R and MC organized the database and performed the statistical analysis. PC-R wrote the first draft of the manuscript that was critically read, revised, and approved by all authors.

### Conflict of interest statement

AO-M is funded by a grant from Schuhfried GmBH for norming and validation of cognitive tests. The remaining authors declare no potential sources of conflict of interest.
