# Supplementary material for: Criterion Validity of the Yale-Brown Obsessive-Compulsive Scale Second Edition for Diagnosis of Obsessive-Compulsive Disorder in Adults
Source: Front Psychiatry. 2018 Sep 11;9:431. doi: 10.3389/fpsyt.2018.00431 (PMC6141833; doi:10.3389/fpsyt.2018.00431)
Supplement: Supplementary file 1 [file Table_1.docx]

**Supplementary Table s1 - Sociodemographic and psychometric data of non-OCD samples.** For all variables, mean and standard deviation are shown, except for gender (presented as percentage of males). ^a^ refers to the statistical comparison (chi-squared or t-test) between the OCD sample and the healthy subjects sample; ^b^ refers to the statistical comparison between the OCD sample and the sample of mood and anxiety disorders; ^c^ refers to the statistical comparison between the sample of healthy subjects and the sample of mood and anxiety disorders.

OCD = Obsessive-compulsive disorder; Y-BOCS-II = Yale-Brown Obsessive-Compulsive Scale-II; BDI = Beck Depression Inventory; STAI = State-Trait Anxiety Inventory; COI = Coimbra Obsessive Inventory

| **Sample** | **OCD (n=52)** | | **Non-OCD: Healthy (n=117)** | | **Non-OCD: Clinical**  **(n=18)** | |  |
| --- | --- | --- | --- | --- | --- | --- | --- |
|  | Range | Mean (SD) | Range | Mean (SD) | Range | Mean (SD) | p-value |
| Gender (percentage of males) | 42.3% | | 29.1% | | 38.9% | | 0.09ª  0.8^b^  0.4^c^ |
| Age (years) | 19-62 | 40.0 (10.0) | 21-57 | 30.9 (7.0) | 20-64 | 46.3 (12.6) | <0.001^a^  0.04^b^  <0.001^c^ |
| Education (years completed) | 7-23 | 14.7 (3.4) | 9-23 | 15.9 (2.4) | 4-21 | 12.3 (5.6) | 0.024^a^  0.09^b^  0.01^c^ |
| Y-BOCS-II total score | 0-45 | 22.7 (10.4) | 0-25 | 1.7 (3.8) | 0-16 | 2.4 (4.6) | <0.001^a^  <0.001^b^  0.53^c^ |
| BDI total score | 1-45 | 22.2 (13.6) | 0-26 | 3.6 (4.6) | 4-42 | 22.8 (12.8) | <0.001^a^  0.88^b^  <0.001^c^ |
| STAI-state score | 22-75 | 47.9 (14.9) | 20-62 | 31.5 (8.0) | 27-75 | 49.3 (13.6) | <0.001^a^  0.75^b^  <0.001^c^ |
| STAI-trait score | 26-77 | 56.9 (14.4) | 20-54 | 30.1 (7.4) | 26-74 | 50.6 (11.6) | <0.001^a^  0.11^b^  <0.001^c^ |
| COI total score | 18-332 | 137.9 (82.7) | 0-96 | 24.8 (20.0) | 7-290 | 73.4 (83.2) | <0.001^a^  0.01^b^  0.03^c^ |
