# Supplementary material for: Criterion Validity of the Yale-Brown Obsessive-Compulsive Scale Second Edition for Diagnosis of Obsessive-Compulsive Disorder in Adults
Source: Front Psychiatry. 2018 Sep 11;9:431. doi: 10.3389/fpsyt.2018.00431 (PMC6141833; doi:10.3389/fpsyt.2018.00431)
Supplement: Supplementary file 2 [file Table_2.docx]

**Supplementary Table s2 – Diagnoses in the clinical non-OCD sample.** Diagnoses were assessed using MINI Neuropsychiatric Interview.

BD = Bipolar Disorder; MDD = Major depressive disorder; GAD = Generalized anxiety disorder; PTSD = Post-traumatic stress disorder.

| **Patient number** | **Diagnosis 1** | **Diagnosis 2** | **Diagnosis 3** |
| --- | --- | --- | --- |
| 1 | MDD – current depressive episode | Agoraphobia |  |
| 2 | BD - Current depressive episode with melancholic features | GAD |  |
| 3 | BD – current depressive episode | GAD |  |
| 4 | MDD – current depressive episode with melancholic features |  |  |
| 5 | GAD | BD (past manic episode) |  |
| 6 | MDD – current depressive episode |  |  |
| 7 | Dysthymia |  |  |
| 8 | MDD – current depressive episode |  |  |
| 9 | MDD – recurrent depressive episode with melancholic features | Panic disorder with agoraphobia | GAD |
| 10 | MDD – current depressive episode | GAD |  |
| 11 | Panic disorder with agoraphobia |  |  |
| 12 | MDD – current depressive episode | PTSD | GAD |
| 13 | MDD – recurrent depressive episode with melancholic features | GAD |  |
| 14 | MDD – current depressive episode | GAD |  |
| 15 | MDD – current depressive episode |  |  |
| 16 | MDD – recurrent depressive episode |  |  |
| 17 | MDD – recurrent depressive episode |  |  |
| 18 | BD - Current depressive episode with melancholic features | GAD | PTSD |
