# Supplementary material for: Criterion Validity of the Yale-Brown Obsessive-Compulsive Scale Second Edition for Diagnosis of Obsessive-Compulsive Disorder in Adults
Source: Front Psychiatry. 2018 Sep 11;9:431. doi: 10.3389/fpsyt.2018.00431 (PMC6141833; doi:10.3389/fpsyt.2018.00431)
Supplement: Supplementary file 3 [file Table_3.docx]

**Supplementary Table s3 – Correlation matrix for psychometric measures in all participants.** Pearson’s product-moment correlation coefficient was used as correlation measure. Significant differences are indicated as: * p<0.05, ** p<0.01 or *** p<0.001.

Y-BOCS-II total = Yale-Brown Obsessive-Compulsive Scale-II; BDI = Beck Depression Inventory; STAI = State-Trait Anxiety Inventory; COI = Coimbra Obsessive Inventory

|  | Y-BOCS-II total | Y-BOCS-II obsessions | Y-BOCS-II compulsions | BDI | STAI-state | STAI-trait | COI |
| --- | --- | --- | --- | --- | --- | --- | --- |
| Y-BOCS-II total | 1 |  |  |  |  |  |  |
| Y-BOCS-II obsessions | 0.96*** | 1 |  |  |  |  |  |
| Y-BOCS-II compulsions | 0.97*** | 0.86*** | 1 |  |  |  |  |
| BDI | 0.57*** | 0.61*** | 0.48*** | 1 |  |  |  |
| STAI-state | 0.43*** | 0.46*** | 0.37*** | 0.84*** | 1 |  |  |
| STAI-trait | 0.68*** | 0.73*** | 0.58*** | 0.86*** | 0.81*** | 1 |  |
| COI | 0.67*** | 0.67*** | 0.64*** | 0.75*** | 0.66*** | 0.75*** | 1 |
